# Associations between intestinal lactic acid bacteria species and feeding habits of zoo animals

**DOI:** 10.20517/mrr.2024.08

**Published:** 2024-06-17

**Authors:** Masanori Horie, Tomoki Ohno, Hitoshi Iwahashi, Maiko Umemura, Kazutoshi Murotomi

**Affiliations:** ^1^Health and Medical Research Institute, National Institute of Advanced Industrial Science and Technology (AIST), Takamatsu 761-0395, Japan.; ^2^United Graduate School of Agricultural Science, Gifu University, Gifu 501-1193, Japan.; ^3^Bioproduction Research Institute, Tsukuba 305-8566 Japan.; ^4^Biomedical Research Institute, National Institute of Advanced Industrial Science and Technology (AIST), Tsukuba 305-8566, Japan.

**Keywords:** Lactobacillaceae, intestinal flora, carnivores, herbivores, piscivores

## Abstract

**Aim:** Lactic acid bacteria are among the most important bacteria in the intestinal flora and often have beneficial effects on the host. It is known that the bacteria that compose the intestinal flora are influenced by the feeding habits of host animals, but there was a lack of knowledge about lactic acid bacteria. Therefore, also considering the use of select strains as probiotics, this study investigated the relationship between the feeding habits of zoo animals and intestinal *Lactobacillaceae* species.

**Methods:** Lactic acid bacteria belonging to the family Lactobacillaceae were isolated and identified from the feces of 20 zoo animal species (5 carnivores, 4 herbivores, 7 piscivores, and 4 omnivores). Isolates were identified by the homology of the 16S rRNA gene sequence. In addition, the fecal flora of host animals was evaluated by the 16S rRNA gene amplicon sequencing.

**Results:** The types of *Lactobacillaceae* species were shown to vary depending on the feeding habits of host animals. *Ligilactobacillus salivarius* (*L. salivarius*) and *Ligilactobacillus saerimneri* (*L. saerimneri*) were isolated from the feces of carnivores. Whereas *Ligilactobacillus equi* (*L. equi*), *Limosilactobacillus gorillae*, *Ligilactobacillus hayakitensis* and *L. salivarius* were isolated from the feces of herbivores. These *Lactobacillaceae* species were not found in the feces of piscivores. Instead, *Enterococcus* were frequently found in piscivores. The fecal flora also differed according to the feeding habits of host animals; at the phylum level, Bacillota was predominant in all animals; on the other hand, herbivores tended to have a higher proportion of Bacteroidota than carnivores, and piscivores tended to have a higher proportion of Proteobacteria.

**Conclusion:** Lactic acid bacteria differ among animal species in a manner dependent on the hosts’ feeding habits.

## INTRODUCTION

Probiotics are defined as “live microbial feed supplement which beneficially affects the host animal by improving its intestinal balance”, a concept proposed by Fuller in 1989^[1]^. Another definition, “mono- or mixed cultures of live microorganisms which, when applied to animal or man, beneficially affect the host by improving the properties of the indigenous microflora”, was also proposed by Havenaar and Huis in’t Veld^[2]^, and Holzapfel *et al*.^[3]^. The Food and Agriculture Organization/the World Health Organization (FAO/WHO) also defined probiotics as “live microorganisms which, when administered in adequate amounts, confer a health benefit on the host”^[4]^_._

Common beneficial effects of probiotics on the host include preventing diarrheal diseases^[5-8]^, stimulating the immune system^[9,10]^, anti-inflammatory effects^[11,12]^, and inhibiting the growth of pathogenic bacteria^[13]^. Other potential benefits of probiotics include regulating blood cholesterol levels, uric acid metabolism^[14]^, promoting skin wound healing^[15]^, reducing abdominal visceral fat and total fat levels^[16]^, and improving liver function^[17]^. Probiotics also affect the intestinal flora. In pigs treated with *Clostridium butyricum* MIYAIRI 588 (CBM588), higher levels of Lactobacillaceae at the family level, *Bifidobacterium* at the order level, and *Lactobacillus ruminis* and *Bifidobacterium pseudolongum* at the species level were observed compared to the non-treated group^[18]^.

The potential of probiotics depends more on the strain than the bacterial species itself. Specific biological activities beneficial to the host are associated with particular bacterial strains. The most important bacteria reported as probiotics are lactic acid bacteria. *Limosilactobacillus reuteri* (*L. reuteri*), as a probiotic, is known to have some beneficial properties to host. *L. reuteri* CRL 1098 reduced blood cholesterol^[19]^. *L. reuteri* ML1 stimulated the immune system^[20]^. Another *L. reuteri* strain eliminated pathogenic microorganisms and fungi by secreting the bacteriocin reuterin^[21]^.

*Limosilactobacillus fermentum* can inhibit the growth of *Candida glabrata* by depleting ergosterol^[13]^. Ingestion of *Lacticaseibacillus casei* (*L. casei*) strain Shirota, also known as Yakult, reduced antibiotic-associated diarrhea^[5]^. *Lactobacillus gasseri* SHMB 0001 derived from human milk showed a protective effect against DSS-induced mice colitis via enforcing gut barrier, downregulating pro-inflammatory cytokines, and modulating gut microbiota^[22]^. Lactic acid bacteria as probiotics exhibit immunostimulatory activity and anti-inflammatory responses through crosstalk with host intestinal epithelium^[23]^. The benefits of probiotics have been observed not only in humans but also in other animals like mammals and birds, particularly in laboratory and domestic animals. For instance, lambs given a mixture of *L. casei* HM-09 and *Lactiplantibacillus plantarum* HM-10 experienced decreased visceral fat accumulation and increased unsaturated fatty acids in muscle while saturated fatty acids decreased^[24]^. Chickens fed *Lacticaseibacillus chiayiensis* AACE3 showed higher body weight gain and digestive enzyme activity, along with lower levels of pro-inflammatory cytokines compared to those not given the probiotic^[25]^.

Administration of a cocktail of lactic acid bacteria consisting of *Lactobacillus crispatus* JB/SL-10, *Limosilactobacillus oris* JB/SL-20, *L. reuteri* JB/SL-25, *Lactobacillus johnsonii* (*L. johnsonii*) JB/SL-39, and *Ligilactobacillus salivarius* (*L. salivarius*) JB/SL-43 improved common enteric disease caused by *Clostridium perfringens* in chickens by improving intestinal morphology and modulating innate immune responses^[26]^. Although these studies were aimed at improving livestock productivity, they show that select lactic acid bacteria used as probiotics are also effective in livestock.

On the contrary, there are typically 500 to 1,000 species of bacteria residing in the intestinal tract, collectively forming the intestinal flora. In the gastrointestinal tract, there are approximately 10^10^-10^12^ cells/g of bacteria inhabiting the large intestine^[3,27]^. Nevertheless, the diversity and abundance of bacteria in the intestine may vary depending on the study. The intestinal flora is intricately linked to the health condition of the host, being influenced by the host’s physiological state.

In recent years, biologists have accumulated evidence for a close relationship between host health and intestinal flora. The intestinal microbiota is affected by the physical condition of the host; for instance, individuals with obesity have a high proportion of Bacillota bacteria. In addition, the intestinal microbiota of individuals without obesity contains a larger proportion of Bacteroidota bacteria than that of individuals with obesity^[28-32]^. The different compositions of these intestinal microbiota can be ascribed to the fact that Bacillota bacteria are energy-efficient and can use sugars in foods that humans cannot digest^[33]^. Diet is the most prominent factor affecting the composition of the intestinal microbiota, including the presence of Lactobacillaceae^[34,35]^. Consuming an animal or plant diet causes changes to the intestinal microbiota that activate the immune system and induce inflammation^[36,37]^. Diets high in saturated fat and animal proteins, such as Western diets, increase the population of Bacteroides in the gut, whereas diets high in dietary fiber and carbohydrates increase the population of *Prevotella*^[38]^. However, individual fixed intestinal flora species fluctuate as a result of short-term dietary changes and general antibiotics^[39]^.

As mentioned earlier, the composition of the intestinal flora varies between individuals consuming animal-based and plant-based diets. When lactic acid bacteria are consumed as probiotics, they do not work in isolation but are believed to influence the existing intestinal flora. This interaction with the intestinal flora is considered one of the key effects of probiotics. However, if inappropriate bacterial species are introduced, they may not survive in the intestinal tract and could prove ineffective as probiotics. Therefore, to optimize the efficacy of lactic acid bacteria as probiotics, it is crucial to combine them with bacterial species naturally present in the intestinal flora. While research on the intestinal flora of humans and laboratory animals, primarily rodents, as well as livestock like pigs, cows, and sheep, is advancing, there is limited knowledge regarding zoo animals.

To maintain the health of zoo animals, lactic acid bacteria supplements are sometimes administered. However, it’s uncertain whether the selected strains are suitable for the host animals. Analysis of the gut microbiota in insectivorous bats, *Nyctalus noctula* (*N. noctula*) and *Vespertilio murinus* (*V. murinus*), and frugivorous bats, *Carollia perspicillata*, revealed notable differences in microbial diversity between the two dietary groups. Conversely, the disparities between *N. noctula* and *V. murinus* were less pronounced. These findings indicate that the gut microbiota of bats is not strictly species-specific but rather influenced by dietary preferences^[40]^.

Choosing the optimal lactic acid bacteria that harmonize with the intestinal flora based on dietary habits is crucial for the health and welfare of zoo animals. Hence, this study aimed to explore the varieties of lactic acid bacteria and the intestinal flora in three common feeding habits among zoo animals: carnivorous, herbivorous, and piscivorous.

## METHODS

### Isolation of lactic acid bacteria from animal feces

Animal feces were provided by the Tobe Zoological Park of Ehime Prefecture (Tobe, Ehime, Japan) and the New Yashima Aquarium (Takamatsu, Kagawa, Japan). Fresh feces that were excreted within 2 days were collected and maintained in chilled storage before isolation. The feces were transferred to sterilized 50-mL centrifuge tubes and mixed with Mitsuoka’s diluted solution (4.5 g of KH_2_PO_4_, 6.0 g of Na_2_HPO_4_, 0.5 g of L-cysteine hydrochloride, 0.5 g of Tween 80, 1.0 g of agar per 1.0 L of distilled water)^[41]^ at a concentration of 0.1 g/mL. The feces were suspended by vortexing. The suspension was diluted serially 103 times with PBS and spread on De Man, Rogosa, and Sharpe (MRS; Merck KGaA, Darmstadt, Germany) or Lactobacillus Selection (LBS; Becton, Dickinson and Company, Franklin Lakes, NJ, USA) agar plates. The plates were incubated at 37 °C for 2 days under anaerobic conditions in an AnaeroPack anaerobic gas chamber (Mitsubishi Gas Chemical Co., Inc., Tokyo, Japan). Single isolated colonies were picked, and individual colonies were inoculated into screw-top test tubes containing 10 mL of MRS broth. Each isolate was incubated at 37 °C for 1-2 days. Stock cultures were stored at 80 °C in MRS broth containing 30% glycerol.

### Identification of lactic acid bacteria

The isolates were identified by homology analysis of 16S rRNA gene sequences. They were cultured overnight in MRS broth and collected by centrifugation at 10,000 × *g* for 10 min. Each pellet was resuspended in elution buffer, and the bacterial cells were lysed by sonication using a Bioruptor (Sonicbio Co. Ltd., Samukawa, Japan). DNA was extracted from the isolates using a DNeasy Blood & Tissue Kit (Qiagen, Hilden, Germany). Polymerase chain reaction (PCR) amplification was performed on an Applied Biosystems Veriti Thermal Cycler (Thermo Fisher Scientific Inc., Waltham, MA, USA) with a set of bacterial universal primers 27F (5’-AGAGTTTGATCCTGGCTCAG‐3’) and 1492R (5’-GGTTACCTTGTTACGACTT‐3’)^[42]^ or 10F (5’-GTTTGATCCTGGCTCA‐3’) and 800R (5’-TACCAGGGTATCTAATCC‐3’) with Takara Ex Taq DNA polymerase (Takara Bio Inc., Kusatsu, Japan). The PCR cycles were conducted with a 50-μL reaction mixture containing 1 μL of template DNA, 1 μmol/L primers, 1.25 U of Ex Taq polymerase, 5 μL of PCR buffer, and 0.2 mmol/L of each deoxynucleotide triphosphate. All primers were synthesized by Eurofins Genomics (Tokyo, Japan). The following thermal cycling conditions were used: initial denaturation for 3 min at 95 °C; 40 cycles of 30 s at 95 °C, 55 s at 55 °C, and 1 min at 72 °C; and a final extension for 10 min at 72 °C. The PCR products were analyzed via electrophoresis in a 1% agarose gel and then stained with ethidium bromide. The primer sequences used for DNA sequencing were as follows: LAB-seqF (5’‐TCCTGGCTCAGGACGAACGCT‐3’) or 10F. Sequencing was performed by Macrogen Japan Corp. (Tokyo, Japan). Sequence identification was performed using the Standard Nucleotide BLAST of the National Center for Biotechnology Information (http://blast.ncbi.nlm.nih.gov/Blast.cgi).

### Evaluation of sugar utilization by lactic acid bacteria

Sugar utilization by the isolates was examined using the API 50 CH system (bioMérieux, Inc., Marcy-I’Etoile, France). The isolates were preincubated in MRS broth overnight and collected by centrifugation at 5,400 × *g* for 10 min. After the bacteria were washed once with PBS, each pellet was resuspended in API 50 CHL medium (bioMérieux) at McFarland Standard No. 2. Each suspension was inoculated in a test tube containing API 50 CH and covered with mineral oil. The bacteria were then cultured for 48 h at 37 °C. Sugar utilization was determined by examining the color of the medium.

### 16S rRNA gene amplicon sequencing and bacterial flora analysis by QIIME2

Genomic DNA for microbiome analysis from animal feces was prepared using ISOSPIN Fecal DNA (Nippon Gene Co., Ltd., Tokyo, Japan) in accordance with the manufacturer’s protocol with a beads cell disrupter (Micro Smash, Tomy Seiko Co., Ltd., Tokyo, Japan). Microbiome analysis based on the V1-V3 region of the 16S rRNA gene was performed by Eurofins Genomics, K.K. (Tokyo, Japan). The reads were filtered using fastp 0.23.2^[43]^. The primer sequences were removed by truncating 19 bases at the 5’ end of the forward read and 23 bases at the 5’ end of the reverse read. The truncation of one base at the 3’ end, removal of reads with an average Q score of less than 30, and truncation of low-quality bases (average Q score of less than 30) at the 3’ end were performed using a sliding window (window size 4). Sequence analysis was performed on the filtered reads using QIIME 2 2022.8^[44]^. Amplicon sequence variants (ASVs) were created using DADA2^[45]^ (q2-Dada2). Bacterial classification was assigned to representative sequences of each ASV using the naive Bayesian classification method with q2-feature-classifier classify-sklearn^[46]^. The V1-V3 regions that had 99% homology to the Silva release 138.1 SSU database were used after processing by q2-feature-classifier fit-classifier-naive-bayes^[47,48]^. Classification was based on Silva release 138.1, instruments were curated by RESCRIPt^[49]^, and extracted V3-V4 regions were based on amplification primer sequences and then processed by qiime feature-classifier fit-classifier-naive-bayes. RESCRIPt curation was performed by removing low-quality sequences (sequences containing more than 5 ambiguous bases or homopolymers longer than 8 bases), base-length filtering (i.e., removing sequences that did not meet the following criteria: archaea ≥ 900 bp, bacteria ≥ 1,200 bp, eukaryotes ≥ 1,400 bp), and deleting redundant sequences. ASVs presumed to be derived from chloroplasts or mitochondria were removed from the summary table.

### Principal coordinate analysis and cluster analysis

Differences between the samples were visualized using Principal Coordinate Analysis (PCoA) and cluster analysis using ASVs. The ASV summary table was diluted to the minimum size using the rarefy function of vegan ver. 2.6-4^[50]^. Representative ASV sequences were aligned using MAFFT ver. 7.490 with the E-INS-i option. Based on the aligned sequences, they were optimized using the GTR + CAT model with fasttree ver. 2.1.11^[50]^. A phylogenetic tree was constructed using the maximum likelihood method. Based on the constructed phylogenetic tree, the unweighted and weighted UniFrac distances between samples were measured using phyloseq ver. 1.38.0 and R ver. 4.2.2^[51,52]^. PCoA was performed using the cmdscale function in R.

## RESULTS

### Isolation of lactic acid bacteria from animal feces

Lactic acid bacteria were isolated from the cultured feces of zoo animals, including carnivores (jaguar, tiger, lion, and puma), piscivores (Asian small-clawed otter, California sea lion, South American sea lion, harbor seal, Baikal seal, and bottlenose dolphin), herbivores [South American tapir, Malayan tapir, horse (Japanese native species: Noma-uma), giraffe, and West Indian manatee], and omnivores (Japanese badger, raccoon dog, and bear). Details of the host animals are shown in Supplementary Table 1. Three species of bear, carnivorous polar bear, herbivorous sun bear, and intermediate Asian black bear, were included. Details of the diet provided to each animal are listed in Supplementary Table 2. Lactic acid bacteria were isolated from the feces of the 20 animals with different eating habits using MRS and LBS agar [Supplementary Table 1]. Former *Lactobacillus* species were frequently isolated from the feces of carnivorous animals. Almost all of the colonies that formed on the MRS and LBS agar plates belonged to the former genus *Lactobacillus*. Differences in the species of lactic acid bacteria among hosts were small. *L. salivarius* was the most frequently isolated, followed by *Ligilactobacillus saerimneri* (*L. saerimneri*). *Enterococcus faecium* was also commonly isolated. Former *Lactobacillus* species were also found in the feces of herbivores; however, they differed from those isolated from the feces of carnivores. *Ligilactobacillus equi* (*L. equi*) and *Limosilactobacillus gorillae* were isolated from the herbivores, and *Ligilactobacillus salivarius* was also isolated from horse feces. The herbivore samples also contained *Weissella confusa* and *Streptococcus* sp. No former *Lactobacillus* species were isolated from giraffe feces. *Ligilactobacillus animalis* *L. animalis* was isolated from the feces of Asian black bear and sun bear, which tend to be herbivorous. By contrast, *Ligilactobacillus agilis* and *L. saerimneri* were isolated from the feces of polar bears. Although the western Indian manatee is an herbivore, no former *Lactobacillus* species were isolated from its feces; instead, the colonies that formed on the MRS agar plates were *Lactococcus garviae* and *E. faecalis*. Similarly, no former *Lactobacillus* species were isolated from the piscivores; instead, *Lactococcus garvieae* (*L. garvieae*), *Streptococcus* sp., and *Enterococcus* sp. were isolated as lactic acid bacteria from these animals.

### Sugar utilization of *Lactobacillaceae* isolated from the carnivores and herbivores

Sugar utilization by *Lactobacillaceae* isolated from the carnivores and herbivores was examined [Supplementary Table 3]. Sugar utilization was more dependent on the bacterial species than on the source of isolation. D-Glucose, D-fructose, N-acetyl glucosamine, and D-sucrose were available in all isolated strains. The types of sugars available to *L. saerimneri* tended to be fewer than those of other bacterial species. Meanwhile, significantly more types of sugars were available for the two strains of *L. saerimneri*, TOB0030 (JCM36439) and TOB1106 (JCM36449). Twenty-three and 19 sugars were available to the *L. saerimneri* strains isolated from lion and polar bear samples, respectively. Galactose was unavailable to most *L. saerimneri* strains, but was available to other species. Furthermore, lactose, melibiose, mannitol and raffinose could not be used in *L. saerimneri*, but many other species could use them. On the other hand, trehalose, which was not available in many strains, was available in all strains of *L. saerimneri*. Among the *L. salivarius* strains isolated from carnivores and herbivores, those isolated from the family Felidae could use 10-14 of the 49 types of sugars examined. By contrast, *L. salivarius* strains isolated from the horse samples used only 16 types of sugar. In particular, *L. salivarius* strains isolated from horse samples were able to use xylitol and D-arabitol.

### Principal coordinate analysis and cluster analysis

**Figure 1:**
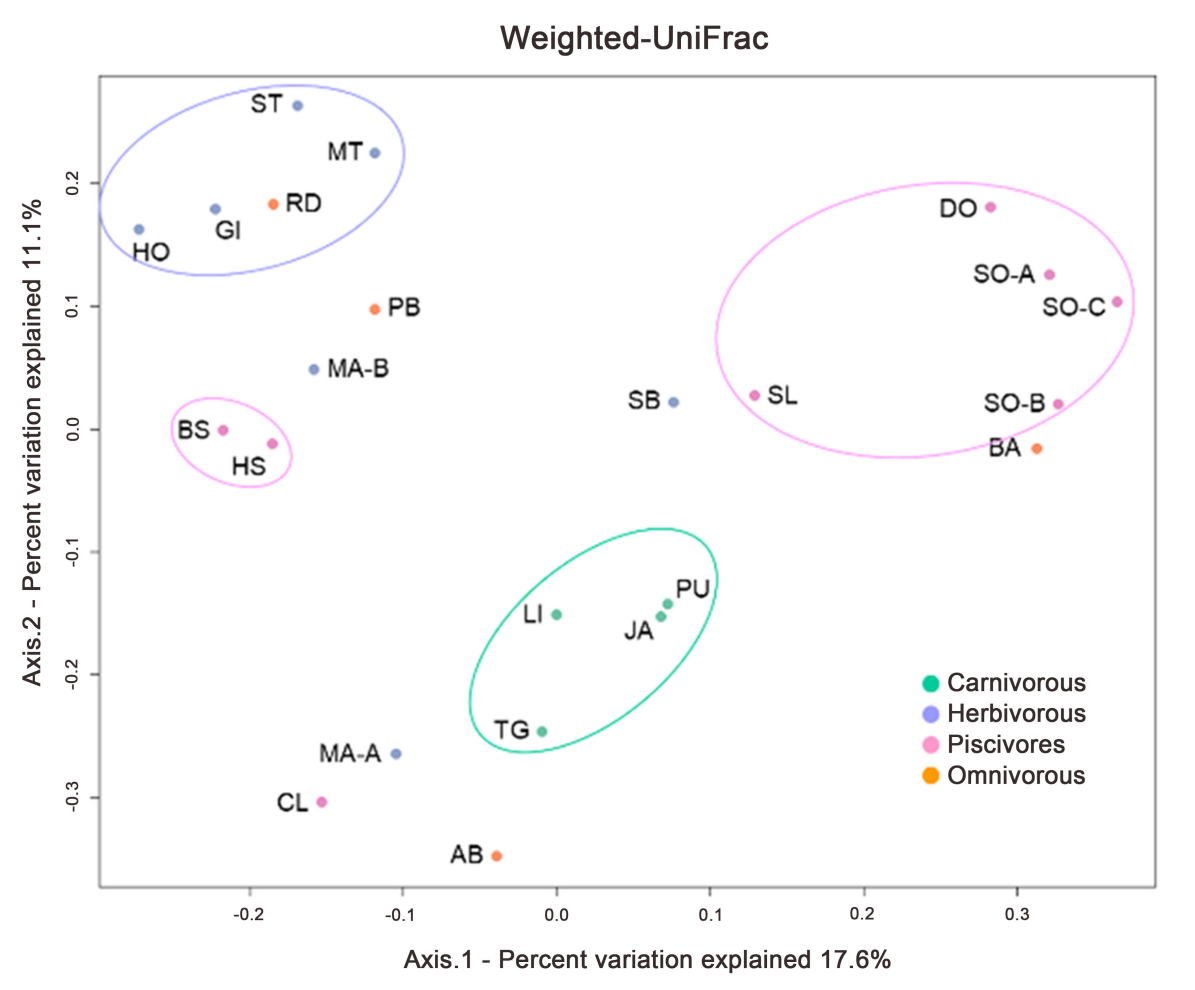
PCoA of the fecal flora of zoo animals. The results of the weighted mean PCoA by genealogical relationship and read number of determined bacteria. PCoA: Principal Coordinate Analysis.

Since the types of lactic acid bacteria tended to differ depending on the feeding habit rather than the species of the host, the intestinal flora was analyzed next. To compare the diversity of the fecal bacterial flora among the animals, UniFrac distance was calculated, and PCoA was performed [Figure 1]. According to unweighted UniFrac analysis, the fecal flora of carnivorous animals showed similarities. The fecal flora of herbivores also showed similarities regardless of whether they were terrestrial, such as the horse, or aquatic, such as the manatee. According to weighted UniFrac analysis, the fecal flora of piscivores showed similarities, in addition to those of carnivores and herbivores. These results suggest that the bacterial components and their proportions in fecal flora depend on the feeding habits of the host animals.

### Fecal floras of the carnivores, piscivores, herbivores, and omnivores

Fecal floras of the carnivores, piscivores, herbivores, and omnivores were analyzed [Supplementary Table 4]. Differences in the composition of fecal flora were observed at the phylum level based on feeding habits. Despite Bacillota being the most dominant regardless of feeding habits, herbivores exhibited a higher proportion of Bacteroidota. The average percentages of Bacteroidota were 7.7%, 58.5%, and 46.2% for carnivores, herbivores, and piscivores, respectively. Conversely, among piscivores, there was a higher proportion of Proteobacteria, followed by Fusobacteriota. At the order level, carnivores showed relatively high proportions of *Peptostreptococcales*-*Tissierellales*. In herbivores, *Bacteroidales* predominated, while *Enterobacterales* and *Fusobacteriales* were more prevalent in piscivores. Additionally, carnivores exhibited a high proportion of *Peptostreptococcaceae* at the family level, along with notable proportions of *Coriobacteriaceae*, *Clostridiaceae*, and *Fusobacteriaceae*. Herbivores displayed a relatively dispersed distribution of bacterial proportions at each level. Similarly, piscivorous animals also showed dispersed proportions, with *Peptostreptococcaceae*, *Oscillospiraceae*, and *Fusobacteriaceae* being relatively dominant. In the case of small-clawed otters, *Fusobacteriaceae* was less prevalent while Enterobacteriaceae was more abundant. At the genus level, *Collinsella*, *Paeniclostridium*, and Fusobacterium were more abundant in carnivores, whereas herbivores exhibited varied bacterial proportions. Piscivores, excluding the small-clawed otter, showed relatively high levels of Bacteroides and Fusobacterium, while *Plesiomonas* was more abundant in small-clawed otters. The fecal flora of animals generally exhibited similar patterns depending on their feeding habits. However, there were some exceptions. For instance, in sun bears, the proportions of *Lactobacillaceae* and *Streptococcaceae* were notably higher, accounting for approximately 80% each. The fecal flora of dolphins tended to differ from those of other animals, and the families *Colwelliaceae* (10.6%), *Flavobacteriaceae* (8.2%), and *Alcaligenaceae* (13.6%) were abundant in these animals. Among omnivorous animals, the fecal flora of Asian black bears and Japanese badgers was relatively similar to that of carnivores, while the fecal flora of raccoon dogs resembled that of herbivores. Although the *Lactobacillaceae* bacteria could be isolated by culture method from the feces of both carnivorous and herbivorous animals, 16S amplicon sequence analysis revealed a low proportion, except in the case of the sun bear. *Turicibacter* was found to be relatively abundant in giraffe, Asian black bear, and Japanese badger flora, with proportions of 3.9%, 6.9%, and 26.9%, respectively.

## DISCUSSION

The lactic acid bacteria species isolated by the culture method tended to differ depending on the feeding habits of the host animals. Furthermore, 16S rRNA gene amplicon sequencing of the feces showed that the composition of the intestinal flora depends on the feeding habits of the host animals. Different lactic acid bacteria were isolated from carnivores and herbivores in this study. *L. salivarius* and *L. saerimneri* were isolated from carnivores, and *L. equi* and *Limosilactobacillus gorillae* were isolated from the herbivores. *L. saerimneri* was also isolated from the feces of the carnivorous polar bear. However, these species were not found in the feces of the herbivorous Asian black bear or sun bear. Instead, *L. animalis* and *Ligilactobacillus murinus* (*L. murinus*) were isolated from these herbivorous bears. The diet of the polar bear consisted of 60% chicken and 12% sausage, whereas that of the sun bear consisted of 60% vegetables, such as steamed potato, apple, and banana. Asian black bears in zoos also tended to be herbivorous, with apples and Chinese cabbage accounting for 40% of their diet. These results suggest that the type of lactic acid bacteria occupying the intestine is more dependent on diet than host species.

The trends in the bacterial composition of the intestinal flora depended more on host feeding habits than on differences in host species. Similar results have been reported in previous studies. A study comparing the gut microbiota of bats in zoos found that while there were significant differences in microbial diversity between the insectivorous species *N. noctula* and *V. murinus* and the frugivorous species *C. perspicillata*, the distinctions between *N. noctula* and *V. murinus* were not statistically significant^[40]^. Among insectivorous bats, the phylum Bacillota was predominant, accounting for approximately 55% of the gut microbiota. In the gut microbiota of frugivorous bats, the phylum Bacillota accounted for 35.7% and Bacteroidota accounted for 30.4%. These results were similar to those of carnivores and herbivores in the present study. On the other hand, at the order level, Lactobacillales were the most dominant among insectivorous bats, which was different from the results of this study. Bacteroidales were predominant (29.9%) in the frugivorous bat, consistent with our results for herbivores.

The presence of species belonging to the former genus *Lactobacillus* in the carnivores was also confirmed through culture method. However, 16S rRNA gene amplicon sequencing did not detect Lactobacillus in tiger, lion, horse, polar bear, or Asian black bear. The DNA extraction method and primer design used to detect *Lactobacillus* species through 16S rRNA gene amplicon sequencing may need to be improved.

The microorganisms that make up the intestinal flora do not exist independently, but rather exist in mutual interference. For example, the metabolites of one microorganism are used by other bacteria, or the production of antibacterial substances such as bacteriocins eliminates similar related species. The microbial environment in the intestine can be considered an ecosystem. When the intestinal flora, which is an ecosystem, is disturbed, it may be effective to use lactic acid bacteria strains that are naturally present there as probiotics in order to restore the original intestinal flora composition.

How are the microorganisms that compose the intestinal flora selected? The specific factors that determine the constituent microorganisms of the intestinal flora have not yet been determined. The most likely potential factor would be the energy source of the bacteria. In general, meat is richer in protein than plants, and plants are richer in dietary fiber. The genus *Paeniclostridium*, found in the fecal flora of carnivores, increased during fermentation using protein and peptone as substrates, and its growth was strongly correlated with the production of ammonium, indole, and p-cresol^[53]^. Analysis of the V4 region of the 16S rRNA gene of healthy domestic ferrets and cats revealed that the fecal microbiota of ferrets exhibited a higher representation of Bacillota and Proteobacteria, including *Clostridium sensu stricto*, *Streptococcus*, *Romboutsia*, *Paeniclostridium*, *Lactobacillus*, *Enterococcus*, and *Lactococcus*^[54]^. On the other hand, Bacteroidota and Actinomycetota were more prevalent in the fecal microbiota of cats. Additionally, in the intestinal flora of dogs fed a 5% chicken liver and heart hydrolysate plus 20% chicken meal diet instead of a 25% chicken meal diet, fecal microbiota was shifted to higher abundance in *Ruminococcus gauvreauii* group as well as lower *Clostridium sensu stricto*, *Sutterella*, *Fusobacterium*, and *Bacteroides*^[55]^. These results suggest that proteins influence the composition of *Paeniclostridium* and *Fusobacterium* in the intestinal flora. Fish-based diets predominantly consist of animal protein, but fecal flora and isolated lactic acid bacteria of piscivorous animals were different from those of carnivorous animals. The factors that determine the intestinal flora of piscivorous animals are still unknown. Analysis of gut microbiota composition in raccoon dogs fed three different diet types (fish and amphibians, mixed protein with maize, and solely maize) exhibited notable variations in the relative abundances of Bacteroidota, Proteobacteria, and Verrucomicrobiota depending on the dietary composition. On the other hand, Bacillota remained the most dominant phylum regardless of feeding habits. Racoon dogs solely fed maize exhibited a significant increase in Proteobacteria, potentially linked to dietary fiber and lignin degradation^[56]^.

The selective pressure acting on the different types of lactic acid bacteria related to the host feeding habits has yet to be clarified. It has been reported that protease activity was high in the fermented liquid of *L. salivarius*, which was found in many carnivorous animals in this study^[57]^. *L. salivarius* may be compatible with carnivores.

Another possibility is the variety of sugar utilization by these bacteria. Herbivore-derived *Lactobacillus* strains tended to rely on more types of sugar than carnivore-derived strains. For example, *L. equi* isolated from the horse could use arabinose, rhamnose, and inulin, which are found in plants. In herbivores, plant-derived polysaccharides may be degraded by other bacteria, and *Lactobacillus* spp., which can use a wide variety of sugars, may be predominant. As an example, genomic analysis of carbohydrate-active enzymes suggested that porcine-derived *L. johnsonii* was capable of utilizing a wide range of carbohydrates^[58]^. However, the origin of lactic acid bacteria living in the intestinal tract and the mechanisms by which they colonize the intestine deserve attention in the future. Nutrient of diet is important for the selection of lactic acid bacteria species. In this study, only the sugar utilization of isolated lactic acid bacteria was investigated. Meat is rich in protein, while fish is abundant in docosahexaenoic acid and eicosapentaenoic acid. To understand why dietary habits exert greater influence on dominant lactic acid bacteria than host species, it is essential to investigate whether these amino acids and fatty acids impact the metabolism and growth of lactic acid bacteria.

Although many types of herbivores were included in the present study, a bias existed in the selection of carnivores. Except for the polar bear, all carnivores included in this study belong to the family Felidae. In this study, we were able to find basic trends in the distribution of intestinal lactic acid bacteria, but the number of samples was insufficient for a complete picture. Thus, other carnivorous families, such as weasel, hyena, wolf, and fox, should be investigated. In addition, herbivores such as ruminant animals may have different Lactobacillus species coexisting in their intestinal tracts that warrant further investigation. In ruminants, metabolism by microorganisms occurs in the rumen; thus, food is digested differently when it reaches the intestinal tract, which could affect the lactic acid bacteria in the flora. In the present study, *Lactobacillus* species were neither found in, nor determined by, 16S rRNA gene amplicon sequencing of the feces of giraffe, a ruminant animal.

Considering the above results, we suggest that the selection of lactic acid bacteria strains used as probiotics should depend on their suitability for the host animals. For example, *L. salivarius* and *L. saerimneri* may be more effective probiotics than *L. equi* in feline carnivores. The administration of *L. equi* to carnivores may not have the desired effect because of their low colonization in the intestine. By contrast, *L. equi* and *L. animalis* may be more effective in herbivores. *Lactobacillus acidophilus* (*L. acidophilus*), which is frequently found in the intestines of humans, was not found in the feces of zoo animals. We did not examine primates in the present study, but the lactic acid bacteria used as probiotics for humans may not be suitable for the zoo animals examined in this study. Although *Lactobacillus* spp. were not found in the feces of the piscivorous or marine animals in this study, *L. salivarius* has been isolated from a rectal swab from a bottlenose dolphin^[59]^. The types of bacteria comprising the intestinal flora of piscivorous or marine animals differed from those in the carnivorous and herbivorous terrestrial animals at the phylum level. Thus, the selection of lactobacilli used as probiotics should be optimized.

## DECLARATIONS

### Availability of data and materials

The isolated lactic acid bacteria strain was deposited at Japan Collection of Microorganisms (JCM), Riken BioResource Research Center. The 16S rRNA gene sequence has been submitted to DDBJ. The datasets generated and analyzed during the current study are available from the corresponding author upon reasonable request.

### Copyright

© The Author(s) 2024.
